# A study on driving behavior characteristics and influencing factors of older drivers at signal-controlled and unsignal-controlled intersections

**DOI:** 10.1371/journal.pone.0326696

**Published:** 2025-06-25

**Authors:** Yang Li, Bingshuo Chen, XiaoHua Zhao, Hongzhen Zhu, Wei Guo, Wei Han

**Affiliations:** 1 Department of Road Traffic Management, Beijing Police College, Beijing, P.R China; 2 Research Institute of Highway, Ministry of Transport, Beijing, P.R China; 3 College of Metropolitan Transportation, Beijing University of Technology, Beijing, P.R China; 4 Functional Safety Department I, Hirain Technologies, Beijing, P.R China; 5 Road Safety Research Center, Beijing Police College, Beijing, P.R China; National Institute of Technology Calicut, INDIA

## Abstract

Due to a decline in psychological function, older drivers have a high incidence of crashes, especially at intersections. The study considered the characteristics of older drivers and designed a driving experiment that includes two scenarios: signal-controlled intersections and unsignal-controlled intersections. A total of 39 drivers participated in the experiment. The results indicated that compared to the young and middle-aged drivers, older drivers exhibited the following characteristics. From a time perspective, older drivers initiated decelerate and turning maneuvers earlier after entering intersections. Their overall turning process was smoother, as indicated by smaller peak steering wheel cornerings, lower steering reversal rates, and reduced lateral acceleration variability. And older drivers slowed down and turned earlier at signal-controlled intersections. From a spatial perspective, older drivers experienced a decrease in speed before entering the two intersections, followed by a sustained increase in speed and steering wheel cornerings. And there were frequent fluctuations in the speed and steering wheel cornering of older drivers at unsignal-controlled intersections. Finally, two Generalized Linear Mixed Models were developed to examine factors affecting driving stability, focusing on speed and steering control. Results showed that traffic control, driver type, and cognitive function had significant impacts. These findings enhance understanding of older drivers’ behavior and provide a reference for improving age-friendly transport systems and safety training. To better support older drivers, vehicle design, traffic signs, infrastructure planning, and management policies should consider their driving characteristics.

## Introduction

The global aging population is rapidly increasing [1]. This trend has been evident in high-income countries such as Japan and Germany for decades ago. As for China, despite entering the aging population for a relatively short time, this issue is gradually becoming prominent. According to the announcement of the 7th National Population Census in 2020, the population aged 60 and above accounted for 18.7%. At the same time, the process of transportation motorization in China is constantly deepening, and the demand for older adults to obtain certificates and drive is increasing. Driving not only allows them to quickly reach their destination anytime and anywhere, but also helps them live a better life. More and more older adults are becoming active drivers [2]. In 2020, the Ministry of Public Security of China lifted the restriction on driving licenses for older adults over 70 years old, resulting in a significant increase in the number of older drivers. At the end of 2020, the number of drivers in China reached 456 million, with 15.306 million older drivers, accounting for 3.36% of all drivers. In terms of growth rate, the average annual increase of older drivers is 1.452 million, a growth rate of 13.1%, which is twice the proportion of driver growth in the same period in the past five years [3]. The Chinese “aging process” of drivers is gradually accelerating. Compared to young people, older people have poorer bones and aged organs, and once injured, their casualties are severe. In 2016, the crash rate, injury rate, and fatality rate of older drivers in China were 12.83 times, 1.72 times, and 2.27 times that of all drivers, respectively. In 2020, this number changed to 9.76 times, 1.8 times, and 2.89 times [3]. Therefore, it is very necessary to study the reasons for the potential unsafe driving of older adults.

Driving requires an appropriate response to the needs of the driving environment, possessing sufficient physical and psychological skills, and driving safely and consistently [4]. It requires drivers to have good perception ability and stable psychological and physical qualities. The cognitive, judgment, execution, reaction speed, and psychological endurance of older drivers experience varying degrees of decline with age [5], resulting in a certain tendency towards dangerous driving. The contradiction between age and driving behavior invisibly makes older drivers a vulnerable group to crashes. The decline in physical strength and endurance of older drivers makes them more prone to fatigue. The decline in vision and hearing makes it difficult to quickly obtain traffic information in complex traffic environments. The weakening of memory and cognitive abilities leads to a decrease to process traffic information [6]. A study suggests that the proportion of road traffic crashes caused by driver cognitive reasons (perception, judgment, and operational errors) is as high as 92% [7]. Therefore, this paper is interested in studying the influence of age and cognitive function factors on the driving behavior of older adults.

Intersections have complex traffic conditions and are typical crash-prone areas. And intersections involve various operations such as braking, stopping, starting, lane changing, and turning. And drivers need to comprehensively analyze multiple types of complex information such as surrounding vehicles, intersection lanes, and signal lights. The probability of crashes occurring when vehicles enter intersections is much higher than that of road sections. Due to the unclear right of way at intersections without signal lights, traffic rules mainly rely on the driver’s observation and judgment of the surrounding environment for driving decisions, which can easily lead to traffic risks. The level of a crash risk at intersections, especially unsigned-controlled intersections, is greatly influenced by the driver’s driving ability [8].

Some scholars have studied common risky driving behaviors among older drivers. Yamani et al. found that older drivers often fail to notice potential threats at intersections. This was mainly manifested in not following parking signs, not being able to stop in front of the stop line, and not being able to recognize potential conflicts with other road users due to not paying attention to intersecting traffic flow [9]. Braitman et al. found that older drivers do not pay attention to the traffic flow on the opposite side when turning left at signalized intersections [10]. Oxley et al. found that older drivers often cannot give way when other vehicles have priority [11]. And compared to middle-aged and young drivers, older drivers had poorer flexibility in gaze transfer modes [12], and lower operational stability and safety at unsignal-controlled intersections [13]. Zhang proposed that when older drivers droved into intersections, their focus was mainly on the area directly in front of the vehicle, lacking observation of the left and right gaze areas [14]. In China, the proportion of crashes occurring at intersections reached 59.7%, with unsignal-controlled intersections accounting for 25% −30% [15]. And the crash rate and fatality rate of older drivers in unsignal-controlled intersections were 8.86 times and 3.87 times higher than that of all drivers, respectively [3]. Therefore, researching the driving behavior characteristics of older drivers at intersections is of great significance for improving their driving safety.

Although prior studies have revealed typical risky behaviors of older drivers at intersections and explored these issues from the perspectives of traffic conflicts and infrastructure cognition, several limitations remain. First, most studies focus on isolated driving errors or eye movement behaviors and lack a dynamic perspective on the “time” and “spatial” of behavioral changes. Second, many studies rely on eye-tracking data or crash statistics, which are insufficient to systematically quantify the relationship between driving behavior metrics and cognitive differences. Third, comparative analyses often use violation-based criteria and do not consider the spatiotemporal variability of older drivers’ operations across different intersection scenarios.

To address these gaps, this study employed a driving simulator experiment and extracts continuous driving behavior data such as vehicle speed and steering angle. It analyzes older drivers’ behavioral characteristics at signal-controlled and unsignal-controlled intersections from both temporal and spatial dimensions. Furthermore, it explores the underlying cognitive mechanisms behind these differences. This comprehensive approach aims to uncover the dynamic process of risky driving behaviors among older drivers and provide theoretical and empirical support for age-friendly intersection design and safety interventions.

## Method

### Ethical review and participant recruitment

This study categorized drivers into groups based on age. Following the Medium- and Long-term Youth Development Plan (2016–2025) and the law of the people’s republic of China on the protection of the rights and interests of older adults [16], young adults in China are defined as individuals aged 14–35 years, while older adults are defined as those aged 60 years or older. Accordingly, in this study, older drivers were defined as those aged 60 years or above, and middle-aged and young drivers were grouped as those aged 18–59 years.

The study was reviewed and approved by the Ethics Committee of Beijing University of Technology on November 29, 2023. Participant recruitment was conducted from December 4, 2023, to December 15, 2023, through an open call for eligible adult volunteers from the general public. Prior to participation, all volunteers were provided with detailed information about the study’s objectives, procedures, and data usage. Each participant voluntarily signed a written informed consent form after reviewing the information. This comprehensive process ensured the study adhered to ethical guidelines and safeguarded participants’ rights and interests.

### Experimental design and steps of driving simulation

#### Route design.

**Fig 1:**
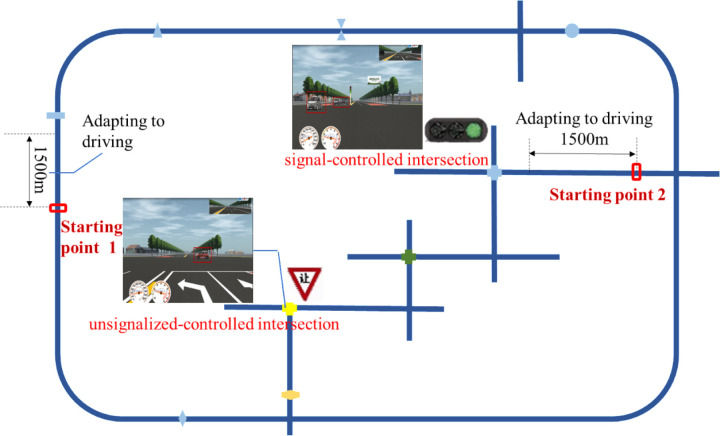
Experimental scenario design.

The route was 16 km long, with one traffic event at each marker, including nine traffic events such as sudden lane change in front of the car, overtaking, driving at the specified speed, sudden passing in front of the bus, children suddenly crossing the motorway, turning left at the signal, turning left at the stop and yield sign, turning left at the slow down and yield sign, and sunbathing. There were road speed limits throughout and each scenario was separated by more than 500m, so there was no mutual interference between the experimental events, and only on the driver learning effect played an interference role. In this study, the yield-controlled sign was selected as the unsignal-controlled intersection, and the events at the signalized and unsignal intersections were demonstrated to investigate the driving behavior characteristics of older drivers at these two intersections. The locations of the intersection event settings relevant to this study are shown in Fig 1.

#### Scene design.

**Fig 2:**
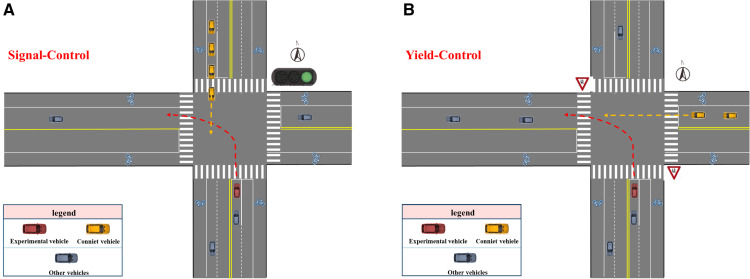
Scenario design for signalized and unsignal-controlled intersection.

Taking into account the typical high-risk scenarios for crashes involving older drivers in China, the study selected two traffic control conditions: unsignal-controlled intersections and signal-controlled intersections. Yield signs were placed before the unsignal-controlled intersections instead of traffic signals. The scenario design is shown in Fig 2.

#### Experimental scale.

Mini mental State Examination (MMSE). MMSE includes five dimensions of test questions: directional ability, memory, attention and computing power, recall ability, and language ability [16]. All were completed in the form of researcher questions and subject responses. The test scores based on correct and incorrect answers to questions. Details of the MMSE are presented in S1 Table in S1 File.

Trail Making Test (TMT). The TMT test is divided into two parts: A and B. TMT aims to measure visual search and sorting, information processing speed, attention distraction, and flexibility [17]. The TMT-A test provides participants with a piece of paper printed with scrambled numbers 1–25, which they need to connect in sequence. The TMT-B test paper includes scrambled numbers 1–13 and letters A-L, which they need to connect in the order of numbers and letters crossing. The test score is the time taken to complete the test correctly. Details of the TMT are presented in S2 Fig in S1 File.

#### Experimental procedure.

Before the official experiment started, a pre-experiment was conducted. Three drivers were arranged to conduct the pre-experiment, including two young and middle-aged drivers and one older driver. Through the pre-experiment, the experimenters were able to familiarize themselves with the experimental process, correct any problems during the experiment and verify the experimental data.

The formal experiment was conducted for 1 test on the above experimental design section with less than 25 minutes of driving time for the road scenario. The details of the formal experiment were as follows:1) Subjects signed an informed consent form to be aware of the experiment information; 2) Subjects filled out a basic information form and four cognitive function scales (MMSE and TMT A/B); 3) A 5-minute driving training was conducted to familiarize the subject with the operation of the steering wheel, gas pedal, and brake pedal; 4) The researcher read out the experimental instructions, including the driving simulation scenario, road types, speed limits, obeying traffic signs, and navigation tips. And the formal experiment started.

During the experiment, subjects were allowed to terminate the experiment on their own for any discomfort. Subjects took approximately one hour to complete the subjective test and driving simulation experiment and were paid approximately ¥100.

### Participants

Determining an appropriate sample size is critical to ensuring the validity of experimental results. In previous studies involving older drivers, the number of participants ranged from 20 to 33 [1,4,18,19]. The determination of the minimum sample size for statistical analysis depends on various factors, including effect size, significance level, and the type of statistical test employed. Therefore, a priori power analysis is commonly used to ensure that the study is sufficiently powered to detect meaningful effects. The sample size should meet the equation (1) [20]. Where $z_{\alpha }/2$ is the upper (α/2)th quantile of standard normal distribution, $z_{\beta }$ is the upper (β)th quantile of standard normal distribution, σ is the standard deviation of normal distribution, ε is the difference between the true mean response of a test factor and a reference value, which given by ε=±δσ, δ is a meaningful difference, in practice, a value between 0.25 and 0.5 was usually chosen [21] if no prior knowledge. Typically, a significance level of less than 10% reflects more than 90% confidence regarding the unknown parameter to avoid Type I error, while a statistical power of more than 80% is chosen to avoid Type II error. Considering the balance of cost-effectiveness, this paper finally chose minimum statistical power (*β* = 0.8), minimum confidence level (*α* = 0.1), and maximum meaningful difference (*δ* = 0.5). Under these assumptions, the minimum required sample size was calculated to be 25. Since our experiment involved 39 participants, the sample size exceeded the minimum requirement, indicating that the study had adequate statistical power to detect meaningful effects.


$$N\;=\;\frac{{\left(z_{\alpha /2}\;+\;z_{\beta }\right)}^{2}\sigma^{2}}{\varepsilon^{2}}$$ (1)

The participants were recruited through multiple channels, including driving schools, community centers, and older driver associations, to ensure diversity in driving experience and social background. This approach ensures the diversity of the sample source and enhances the external validity of the study.

To ensure the validity and reliability of the research data, all participants were required to meet the following criteria: a) Hold a valid driver’s license to ensure legal eligibility for driving; b) Have driven more than 2500 kilometers in the past year and have recent driving experience within the last month to ensure familiarity with driving operations; c) Have normal unaided vision and do not wear glasses during daily activities to ensure clear observation of the driving environment; d) Have normal hearing, capable of accurately hearing traffic sounds and receiving navigation instructions during the driving scenarios; e) In good health and free from simulator discomfort (such as simulator sickness) to ensure the safety of the experiment and the reliability of the data; f) Have no alcohol or other substance addictions to ensure participants are in a sober and normal physical state; g) Have no history of major traffic crashes.

Based on this, the study employed a stratified random sampling method, using age as the stratification criterion to ensure the reasonable distribution of different age groups. Subsequently, within each age group, all individuals who met the screening criteria were assigned the same probability of being selected, and the final list of participants was determined through computer random selection, reducing selection bias and adhering to the principles of random sampling.

### Data analysis and processing

#### Dataset construction.

The comprehensive data sets obtained by subjective questionnaire and driving simulator are as follows: 1) demographic information data, including driver’s age, gender, driving age, driving frequency, etc.; 2) driving behavior data, including main vehicle coordinates, speed, longitudinal acceleration, lateral acceleration, brake pedal and other data output from the driving simulation system.

#### Data pre-processing.

The data pre-processing are as follows: 1) Intercepting the main vehicle behavior data according to the coordinates of the starting point of the event; 2) Converting the original csv file into an excel file through data format conversion to facilitate subsequent indicator extraction.

To explore the driving behavior of the typical intersection turning process of older drivers, the driving behavior characteristics from approaching the intersection to crossing the intersection were fine-grained portrayed. In the spatial dimension, studies had shown that the driver’s speed changed about 150m before the stop line, and the driver’s perceived distance to traffic signs was 200m [22]. Therefore, this study took 200m before the stop line as the starting point for approaching the intersection. In addition, some studies had shown that the driving behavior 100m before the stop line was critical when drivers turned left, so this study took 100m before the stop line as the starting point of the preparatory turn [23,24]. In the temporal dimension, drivers’ behavioral performance in the 10s before and 5s after the stop line when turning was explored.

### The Generalized Linear Mixed Model

The Generalized Linear Mixed Model (GLMM) can be seen as an organic combination of generalized linear models (GLM) and linear mixed models [25]. It is based on the GLM framework and introduces random effects, making it capable of handling data with complex related structures, such as longitudinal data and repeated measures. Additionally, GLMM is well-suited to handle non-normally distributed data, such as overdispersed data commonly seen in driving behavior measurements, as well as potential autoregressive or heteroscedastic structures within the data [26–28].

The general expression of GLMM is as follows:


Y=Xβ+Zμ+ε (2)

where X is the known fixed effects design matrix, Z is the design matrix for the random effects, β is the vector of unknown regression coefficients, representing fixed effects, μ is the vector of random effects parameters, ε is the vector of random errors.

In this study, driving behavior data were collected across multiple trials per participant, leading to within-subject correlations that a traditional linear model might not adequately handle. GLMM allows us to model these dependencies, thereby improving the robustness and accuracy of our statistical inferences. This study takes the influencing factors (traffic control facilities, drivers, cognitive abilities (MMSE and TMT-A/B)) as fixed and random effects, and uses Speed variation and Steering wheel cornering variation as dependent variables to conduct GLMM analysis to explore the influencing factors of driving behavior.

## Results

### Subject demographic information

A total of 39 subjects (20 young and middle-aged drivers and 19 older drivers) were recruited to participate in the study. The gender ratio distribution was roughly in line with the current statistical characteristics of Chinese drivers [20]. The demographic data of all participants are shown in Table 1.

**Table 1:** Descriptive statistics of driver characteristics.

Drivers		Description	Number	M	SD
**Older drivers**	Gender	Man	15	–	–
		Woman	5	–	–
	Age	(60–73) years old	–	66	3.8
	Driving experience	(13–49) years	–	30	8.8
**Young and middle-aged drivers**	Gender	Man	14	–	–
		Woman	5	–	–
	Age	(20–54) years old	–	32	11
	Driving experience	(1–25) years	–	7.2	6.1

### Driving behavior characteristics of different ages drivers in the time dimension

#### Overall difference.

This study investigated the driving behavior characteristics of older drivers from a human factors perspective. It focused on the indicators selected by drivers during the turning process at intersections. The driver’s speed and steering wheel cornering were constantly changing when the driver turns left at the intersection. Therefore, speed and steering wheel cornering was selected as longitudinal and lateral driving behavior indicators, respectively.

As shown in Table 2, in the time dimension, older drivers had higher speeds and lower steering wheel cornering at the two intersections, although this difference was not statistically significant (p = 0.170, p = 0.918, p = 0.642, p = 0.427). Although the overall analysis did not show significant differences, this result provides a global perspective on the overall performance of driving behaviors in both groups. To further explore the changes in driving behavior when approaching intersections, the study will further investigate the differences at different time points.

**Table 2:** Differences in driving behavior of older drivers and other drivers (Time).

Intersection	Drivers	Speed			Steering wheel cornering		
		M (km/h)	SD (km/h)	Sig.	M (°)	SD (°)	Sig.
Signal-controlled	Older	18.329	5.221	0.170	43.792	10.865	0.918
	Young and middle-aged	16.334	3.280		44.138	9.259	
Unsignal-controlled	Older	24.176	6.841	0.642	46.370	13.081	0.427
	Young and middle-aged	23.112	6.967		54.701	42.099	

#### Time point-by-point characteristics at the signal-controlled intersection.

**Fig 3:**
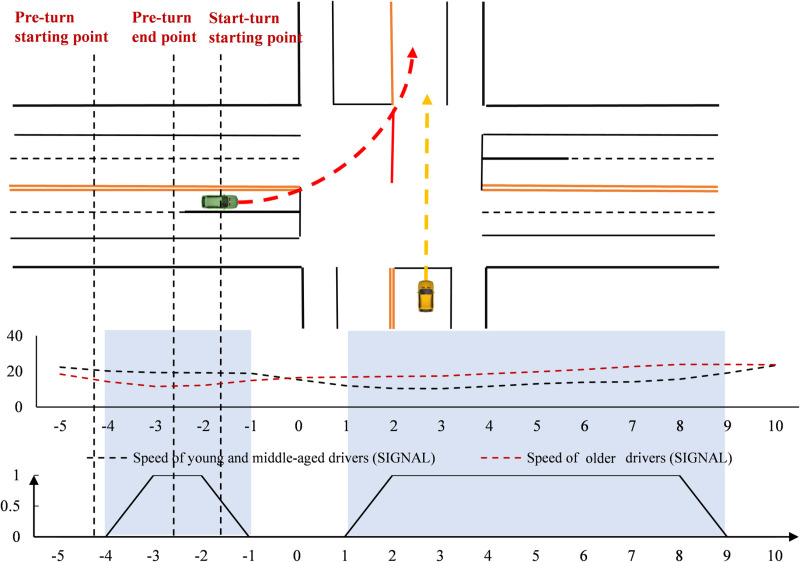
Speed variation at signal intersections (Time).

The speed of drivers was analyzed over time (Fig 3). At the signal-controlled intersection, young and middle-aged drivers drove faster than older drivers before the turn, though the difference was small. After the turn, their speed was slightly lower. In terms of point-by-point spatial significance, there were multiple time points showing significant differences between older and younger/middle-aged drivers. These occurred both immediately after the start of the turn preparation and after completing the turn.

**Fig 4:**
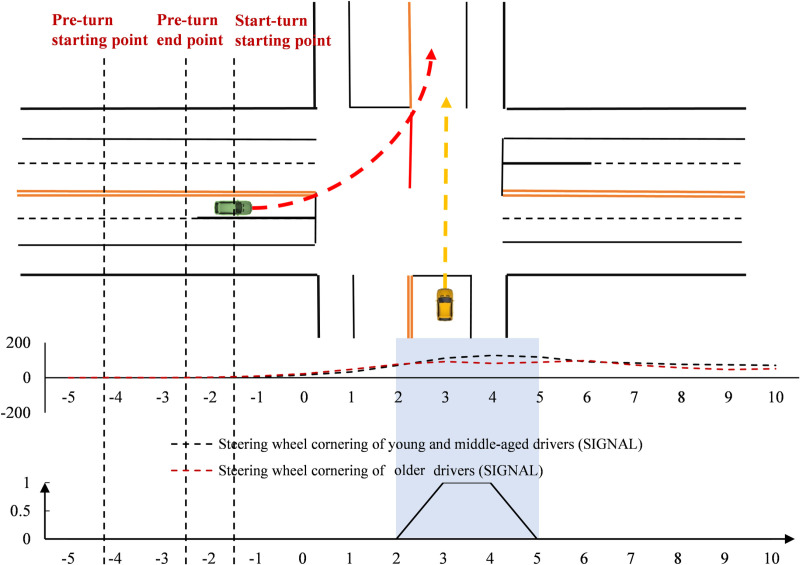
Steering wheel cornering variation change at the signal-controlled intersection (Time).

The steering wheel turning angles were analyzed over time (Fig 4). At the signal-controlled intersection, both groups began steering at nearly the same time. However, older drivers showed faster changes, while young and middle-aged drivers had smoother steering. Both groups reached their peak turning angles after the stop line, but older drivers peaked earlier, and middle-aged drivers had a higher peak angle.

#### Time point-by-point characteristics at the unsignal-controlled intersection.

**Fig 5:**
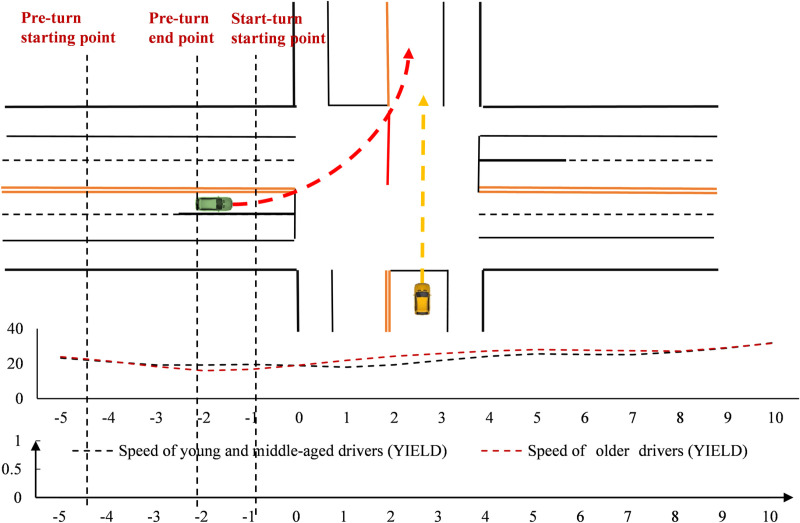
Speed variation at the unsignal-controlled intersection (Time).

Driver speeds were analyzed over time (Fig 5). At the unsignal-controlled intersection, two groups had similar speeds during the turn. However, young and middle-aged drivers were slightly faster before the turn and slightly slower after. In terms of point-by-point spatial significance, there were no significant differences between the two groups at any time during the turning process.

**Fig 6:**
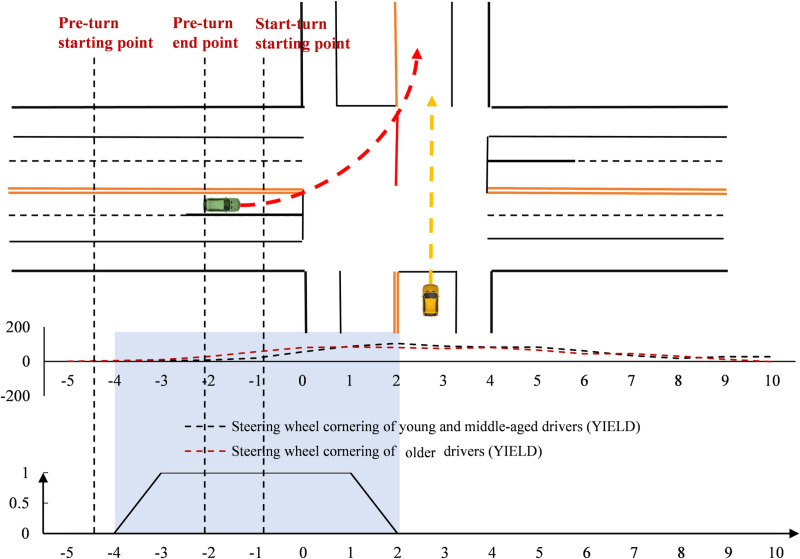
Steering wheel cornering variation at the unsignal-controlled intersection (Time).

The steering wheel turning angles of drivers were analyzed in the time dimension (Fig 6). At the unsignal-controlled intersection, older drivers started steering first, but with a faster steering amplitude. In contrast, young and middle-aged drivers had a smoother steering amplitude. Older drivers reached their peak near the stop line, but the peak was smaller. Young and middle-aged drivers reached their peak after 2 seconds past the stop line. However, point-by-point significance analysis did not show a significant difference.

### Driving behavior characteristics of different ages drivers in the spatial dimension

#### Overall difference.

As shown in Table 3, older drivers had lower speeds at both intersections. At the signal-controlled intersection, their speed was significantly lower than that of young and middle-aged drivers (p = 0.002). Their steering wheel turning angle was smaller at the signal-controlled intersection but significantly larger at the unsignal-controlled intersection (p = 0.001).

**Table 3:** Differences in driving behavior of older drivers and other drivers (Space).

Intersection	Drivers	Speed			Steering wheel cornering		
		M (km/h)	SD (km/h)	Sig.	M (°)	SD (°)	Sig.
Signal-controlled	Older	25.534	4.328	0.002	25.534	4.328	0.835
	Young and middle-aged	31.652	6.519		31.652	6.519	
Unsignal-controlled	Older	26.604	5.708	0.152	31.403	1.617	0.001
	Young and middle-aged	29.374	5.806		28.628	2.772	

#### Space point-by-point characteristics at the signal-controlled intersection.

**Fig 7:**
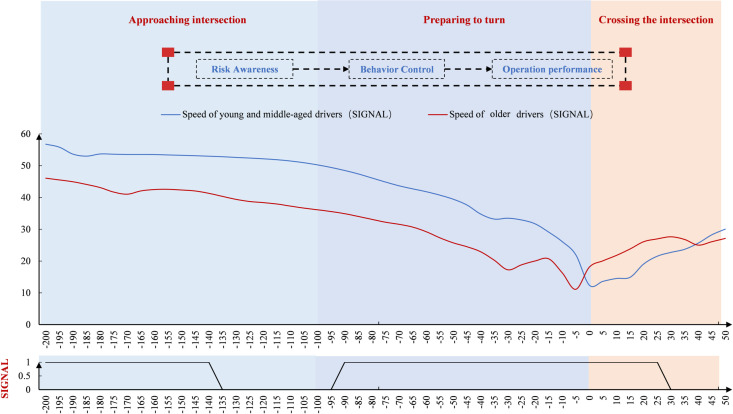
Driver speed variation at the signal-controlled intersection (Space).

Driver speed was analyzed in the spatial dimension (Fig 7). Speed gradually decreased after −200 m, then declined more sharply after 100 m as drivers prepared to turn. Around 0 m, speed began to increase. Both groups slowed down before 0 m, reaching their minimum speed just before the intersection. Notably, there was a slight upward trend before 0 m, and after 0 m, the speed of young and middle-aged drivers showed greater fluctuation. In terms of point-by-point spatial significance, significant differences between the two groups began to appear 65 m before the intersection and continued through the turning and crossing processes.

**Fig 8:**
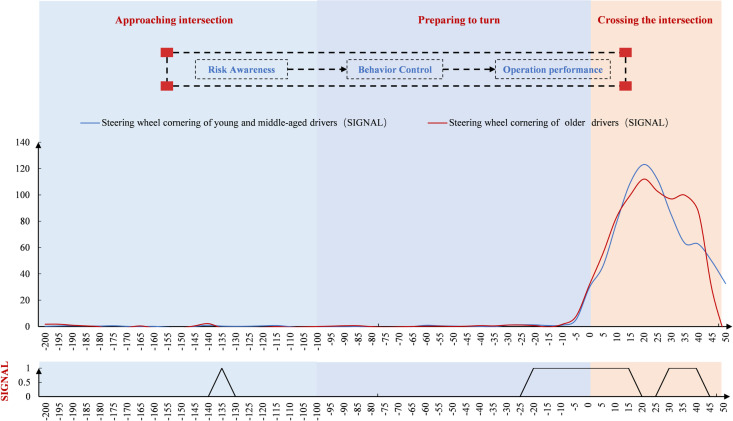
Driver steering wheel cornering change at the signal-controlled intersection (Space).

Fig 8 shows that drivers’ steering wheel cornerings remained stable around 0 before −10 m. After −10 m, the angles changed significantly—first increasing, then decreasing. At signal-controlled intersections, both age groups showed similar increases in steering speed, with peak angles occurring during the crossing. Younger and middle-aged drivers reached higher peak angles, while older drivers exhibited greater fluctuations. Significant spatial differences between age groups were observed before, during, and after the intersection. Older drivers showed more variability across measurement points.

#### Space point-by-point characteristics at the unsignal-controlled intersection.

**Fig 9:**
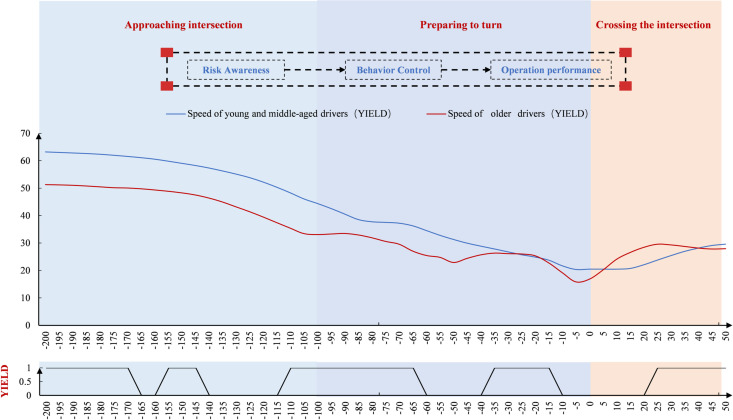
Driver speed variation at the unsignal-controlled intersection (Space).

Driver speed was analyzed in the spatial dimension (Fig 9). Speed gradually decreased after −200 m, then declined more steeply, and slowed again beyond 100 m. Near 0 m, speed began to increase. Older drivers showed a sharper speed reduction before 0 m and faster acceleration afterward compared to young and middle-aged drivers. Both groups reached their minimum speeds before 0 m. At unsignalized intersections, significant differences in turning behavior were more scattered in older drivers, especially concentrated at multiple spatial points during turn preparation.

**Fig 10:**
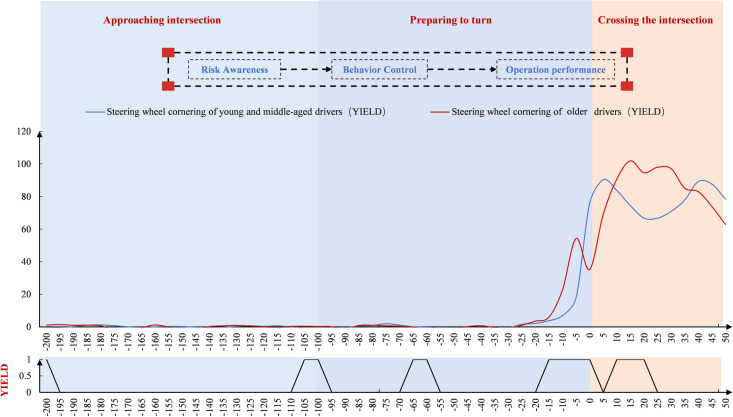
Driver’s steering wheel cornering change at the unsignal-controlled intersection (Space).

As shown in Fig 10, steering wheel cornerings remained stable around 0 before −20 m. After −20 m, the angles changed significantly, first increasing and then decreasing. Compared to young and middle-aged drivers, older drivers showed a faster and less stable rise in steering angle at the unsignal-controlled intersection. Both groups reached their peak angles during the intersection, with older drivers peaking earlier and at a higher value. Older drivers also exhibited greater fluctuations while crossing. In terms of spatial point-by-point significance, notable differences emerged during the steering preparation phase, with more differences observed before and after the intersection.

### Driving behavior of older drivers in the two intersections

#### Overall difference.

As shown in Table 4, in the temporal dimension, older drivers had significantly higher speeds at unsignal-controlled intersections (p = 0.007). At signal-controlled intersections, older drivers had larger steering wheel turn angles, though the difference was not statistically significant. In the spatial dimension, the mean speed of older drivers was higher at unsignal-controlled intersections, but the difference was not statistically significant. However, their steering wheel turn angle was significantly larger at unsignal-controlled intersections (p = 0.015).

**Table 4:** Intersection driving behavior differences for older drivers.

Dimension	Intersection	Speed			Steering wheel cornering		
		M (km/h)	SD (km/h)	Sig.	M (°)	SD (°)	Sig.
**Time**	Signal-controlled	18.329	5.221	0.007	46.370	13.085	0.525
	Unsignal-controlled	24.176	6.841		43.792	10.865	
**Space**	Signal-controlled	25.534	4.328	0.530	29.121	3.416	0.015
	Unsignal-controlled	26.605	5.708		31.403	1.617	

#### Differences in the time dimension.

**Fig 11:**
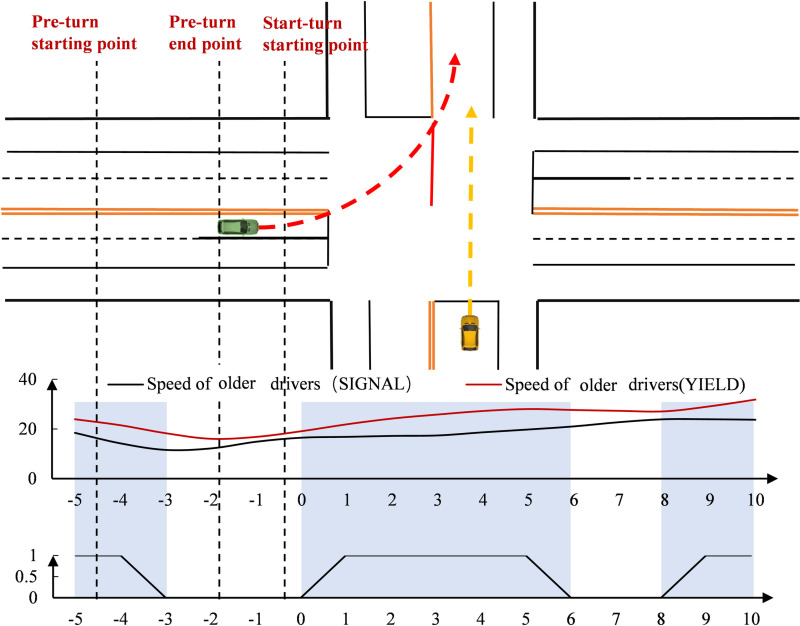
Differences in speed changes of older drivers (Time).

As shown in Fig 11, older drivers at unsignal-controlled intersections were faster, with the smallest speed difference between the two intersections occurring in the 2s range before the stop line. The speed change trend for older drivers was the same at both intersections before the stop line, but faster at unsignal-controlled intersections after the stop line. Additionally, the speed variation of older drivers at signal-controlled intersections was smaller and more stable. In terms of point-by-point significance, significant speed differences between older drivers at signal-controlled and unsignal-controlled intersections appeared at multiple points after the stop line.

**Fig 12:**
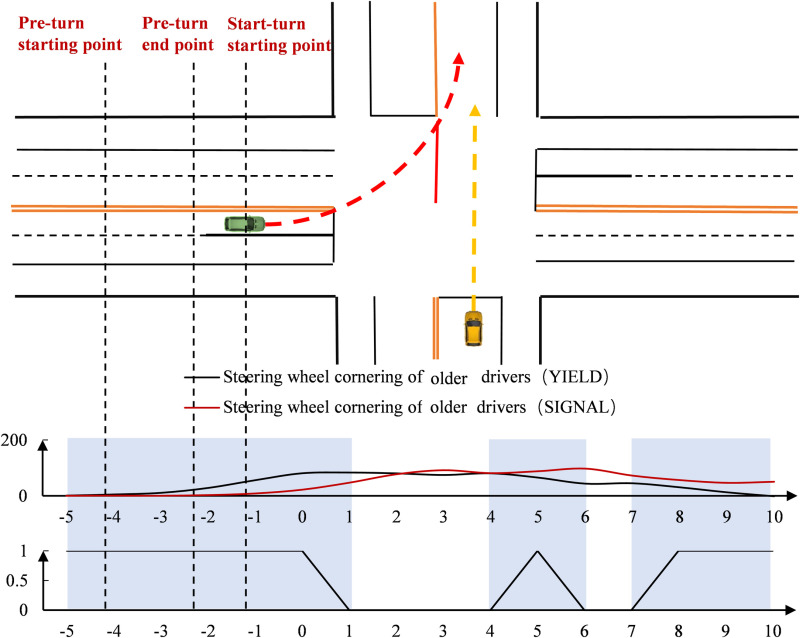
Differences in steering wheel cornering changes of older drivers (Time).

As shown in Fig 12, regarding the change in steering wheel cornering, older drivers at unsignal-controlled intersections had slightly higher angles before 2s and lower angles after 2s. Their steering wheel cornering started to increase earlier and decrease sooner compared to other drivers. The difference in peak steering wheel cornerings between older drivers at the two intersections was not significant, but the change in steering wheel cornering occurred more rapidly at signal-controlled intersections. In terms of point-by-point significance, significant differences in steering wheel cornerings for older drivers were more concentrated before the stop line and also appeared at multiple time points starting 7s after the stop line.

#### Differences in the spatial dimension.

**Fig 13:**
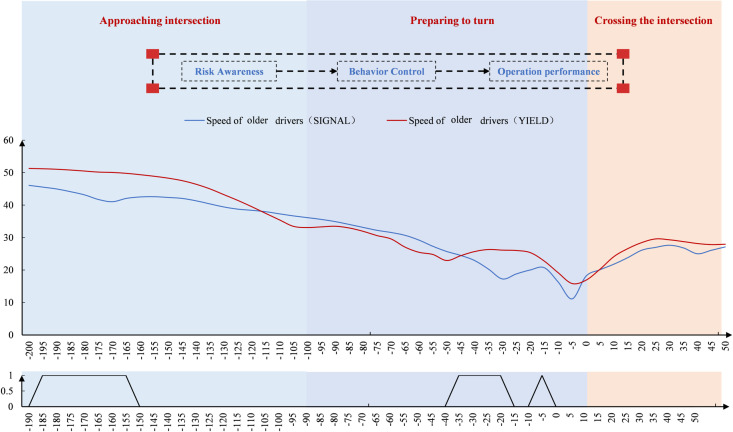
Differences in speed changes of older drivers (Space).

As shown in Fig 13, when observing speed changes in the spatial dimension, speed at both intersections initially decreased and then increased. In the area approaching the intersection, the speed of older drivers at signal-controlled intersections was slightly lower. In the ready-to-turn area, their speed was slightly higher at signal-controlled intersections, but after 45m before the stop line, the speed of older drivers at unsignal-controlled intersections increased, surpassing the speed at signal-controlled intersections. Overall, the speed change of older drivers at unsignal-controlled intersections fluctuated more and showed greater instability. In terms of spatial point-by-point significance, significant differences in speed were observed within 40m of approaching the intersection and at multiple spatial points near the stop line between older drivers at signal-controlled and unsignal-controlled intersections.

**Fig 14:**
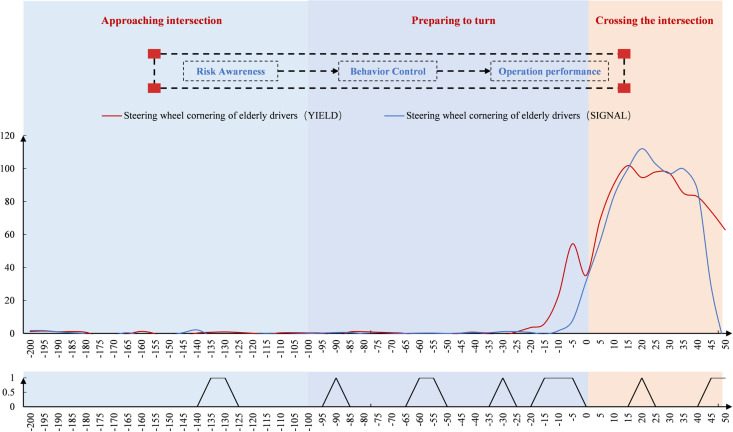
Differences in steering wheel cornering changes of older drivers (Space).

As shown in Fig 14, when observing the change in steering wheel cornering in the spatial dimension, the steering wheel cornering at both unsignal-controlled and signal-controlled intersections first increased and then decreased. In the approaching intersection area, there was no significant change in the steering wheel cornering of older drivers at either type of intersection. However, after entering the ready-to-turn area, 25m before the stop line, older drivers at unsignal-controlled intersections began to increase their steering wheel cornering. The peak angle occurred in the steering area, with the peak at signal-controlled intersections being greater. Overall, the steering wheel cornering changes of older drivers at unsignal-controlled intersections fluctuated more, while the changes at signal-controlled intersections were faster. In terms of spatial point-by-point significance, more significant differences in steering wheel cornerings between older drivers at signal-controlled and unsignal-controlled intersections were found within the ready-to-steer and steering areas.

### Factors influencing cognitive response of older drivers

#### Variables related to cognitive responses.

The previous section provided a detailed analysis of the speed and steering wheel cornering characteristics of older adults when driving through intersections, showing that both behaviors exhibited fluctuations and instability. To explore the underlying mechanisms of these behaviors, this paper further investigates the factors influencing speed and steering wheel cornering fluctuations, as these indicators reflect the longitudinal and lateral stability of driving.

The GLMM method was used to identify the factors influencing driving behavior. The variable information is presented in Table 5. The dependent variables were driving behavior, specifically the standard deviations of speed and steering wheel cornering, which were used to assess driving stability. The independent variables included traffic control conditions, driver type, and four cognitive function scores.

**Table 5:** Factors influencing cognitive response.

Variables	Subclassification
**Traffic control conditions**	Signal-controlled intersections/Unsignal-controlled intersections
**Driver type**	Older drivers/Young and middle-aged drivers
**MMSE**	Cognitive normal/Cognitive impairment
**TMT-A**	Cognitive normal/mild cognitive impairment/Cognitive impairment
**TMT-B**	Cognitive normal/Cognitive impairment

#### Construction of Generalized Linear Mixed Model.

Two generalized linear mixed-effects models were constructed for three typical cognitive driving behavior indicators in the driving simulation test: speed standard deviation and steering wheel cornering standard deviation. As shown in Table 6, the covariance structure with the smallest AIC and BIC values was selected to build the models. The chosen covariance type for both models was Diagonal.

**Table 6:** Selection of covariance types.

Covariance type	Fit measure	Speed standard deviation	Steering wheel cornering standard deviation
**Variance components**	AIC	468.312	453.728
	BIC	482.541	467.982
**Autoregressive moving average**	AIC	472.689	459.214
	BIC	487.015	472.801
**Compound symmetry**	AIC	461.237	448.905
	BIC	475.368	462.732
**Unstructured compound symmetry**	AIC	470.458	457.631
	BIC	484.712	471.524
**Diagonal**	AIC	455.726	443.219
	BIC	469.805	456.837
**Standard identity**	AIC	478.532	464.897
	BIC	492.671	479.012
**Toeplitz**	AIC	474.815	461.328
	BIC	488.904	475.629
**Unstructured**	AIC	489.173	476.581
	BIC	503.412	490.768

Table 7 summarizes the main variables and interaction variables that have a significant impact on driving behavior stability. Specifically, time-to-collision shows significant differences across different classifications of Traffic control conditions (β = 6.015), Traffic control conditions * Driver type (β = 3.410), and Driver type * TMT-A (β = 2.682). Similarly, steering wheel cornering standard deviation exhibits significant differences across various classifications of Traffic control conditions (β = 12.984), Traffic control conditions * Driver type (β = 5.226), Traffic control conditions * TMT-A (β = 2.226), Traffic control conditions * TMT-B (β = 2.817), and Driver type * MMSE (β = 2.965). The fixed-effect solutions of the model, as presented in Table 8, further quantify the differences in the impact of different classifications of independent variables on the dependent variables.

**Table 7:** Fixed effects of driving behavior stability.

Variables	Speed standard deviation		Steering wheel cornering standard deviation	
	F	P	F	P
**Traffic control conditions**	6.015^***^	0.004	12.984^***^	0.000
Traffic control conditions * Driver type	3.410^*^	0.065	5.226^**^	0.039
Traffic control conditions *MMSE	0.106	0.899	1.990	0.144
Traffic control conditions *TMT-A	1.550	0.196	2.226^*^	0.074
Traffic control conditions *TMT-B	1.828	0.167	2.817^*^	0.066
Driver type *MMSE	0.416	0.521	2.965^*^	0.089
Driver type *TMT-A	2.682^*^	0.074	0.582	0.561

Note:

* p < 0.1;

** p < 0.05;

*** p < 0.01.

**Table 8:** Fixed-effect solutions of driving behavior stability.

Variables		Speed standard deviation		Steering wheel cornering standard deviation	
		*β*	p	*β*	p
**Traffic control conditions**	Signal-controlled intersections (vs Unsignal-controlled intersections)	4.446^**^	0.033	14.253^***^	0.001
**Driver type**	Older drivers (vs Young and middle-aged drivers)	−2.755	0.361	9.051^*^	0.065
**MMSE**	Cognitive normal (vs cognitive impairment)	−1.117	0.414	−1.664	0.510
**TMT-A**	Cognitive normal (vs Cognitive impairment)	−1.103	0.544	0.959	0.766
	Mild cognitive impairment (vs Cognitive impairment)	−4.119^***^	0.002	0.253	0.916
**TMT-B**	Cognitive normal (vs Cognitive impairment)	−0.776	0.587	−1.041	0.693
Traffic control conditions * Driver type	Signal-controlled intersections * Older drivers (vs Signal-controlled intersections * Young and middle-aged drivers)	−1.973^*^	0.064	−2.408	0.519
Traffic control conditions * MMSE	Signal-controlled intersections * Cognitive normal (vs Signal-controlled intersections* Cognitive impairment)	−0.988	0.65	−7.941^*^	0.061
	Unsignal-controlled intersections * Cognitive normal (vs Unsignal-controlled intersections* Cognitive impairment)	−0.328	0.881	−0.026	0.995
Traffic control conditions * TMT-A	Signal-controlled intersections * Cognitive normal (vs Signal-controlled intersections * Cognitive impairment)	−1.234	0.593	−10.098^**^	0.023
	Signal-controlled intersections * Mild cognitive impairment (vs Signal-controlled intersections * Cognitive impairment)	−3.804^*^	0.059	−10.036^**^	0.012
Traffic control conditions * TMT-B	Signal-controlled intersections * Cognitive normal (vs Signal-controlled intersections * Cognitive impairment)	3.922^*^	0.068	9.597^**^	0.023
Driver type * MMSE	Older drivers * Cognitive normal (vs Older drivers * cognitive impairment)	−1.657	0.521	−6.904^*^	0.089
Driver type * TMT-A	Older drivers * Cognitive normal (vs Older drivers * Cognitive impairment)	−1.183	0.643	−3.339	0.405
	Older drivers * Mild cognitive impairment (vs Older drivers * Cognitive impairment)	3.405	0.126	−3.684	0.300
Driver type * TMT-B	Older drivers * Cognitive normal (vs Older drivers * Cognitive impairment)	3.403	0.127	1.429	0.702

Note:

* P < 0.1;

** P < 0.05;

*** P < 0.01.

***Speed standard deviation model*:** In terms of the main effects of factors, when considering unsignal-controlled intersections as the reference category, the speed standard deviation of drivers at unsignal-controlled intersections (β = 4.446) was significantly larger, indicating greater speed fluctuations in these environments. Using drivers with cognitive impairment as the reference category, the speed standard deviation of drivers with TMT-A mild cognitive impairment (β = −4.119) was significantly smaller, suggesting that these drivers maintained a more stable speed compared to those with more severe cognitive impairment.

Regarding the interaction effects, taking signal-controlled intersections * young and middle-aged drivers as the reference category, signal-controlled intersections * older drivers (β = −1.973) had significantly lower speed standard deviation, implying that older drivers exhibited more stable speed control at signal-controlled intersections than their younger counterparts. Similarly, drivers with TMT-A cognitive impairment (β = −3.804) had significantly smaller speed standard deviations at signal-controlled intersections compared to those with mild cognitive impairment, suggesting that those with greater cognitive deficits exhibited more conservative speed variations. However, this trend was reversed for TMT-B cognitive impairment (β = 3.922), where these drivers demonstrated greater speed fluctuations, indicating a different impact of cognitive impairment type on speed control.

***Steering wheel cornering standard deviation model*:** In terms of the main effects of factors, when considering unsignal-controlled intersections as the reference category, drivers at signal-controlled intersections (β = 14.253) exhibited a larger standard deviation in steering wheel cornering, suggesting that navigating signal-controlled intersections required more frequent or larger steering adjustments. Using young and middle-aged drivers as the reference category, older drivers (β = 9.051) had a greater standard deviation in steering wheel cornering, indicating that they experienced more variability in lateral control.

For the interaction effects, MMSE cognitive normal drivers (β = −7.941), TMT-A cognitive normal drivers (β = −10.098), and TMT-A mild cognitive impairment drivers (β = −10.036) had smaller steering wheel cornering standard deviations compared to cognitively impaired drivers at signal-controlled intersections, suggesting that these groups maintained more stable lateral control. However, TMT-B cognitive normal drivers (β = 9.597) exhibited a larger standard deviation, indicating that they made more frequent or larger steering adjustments. Additionally, MMSE cognitive impairment older drivers (β = −6.904) had a smaller standard deviation in steering wheel cornering, implying more stable lateral control compared to other impaired groups.

## Discussion

This study explored the driving behavior characteristics of both young and middle-aged drivers and older drivers at the two intersections. The findings contribute to a better understanding of the driving behavior of older adults, providing a foundation for optimizing traffic facilities and conducting safety education and training.

### Differences of different ages drivers in the time dimension

**In terms of longitudinal driving behavior,** older drivers reduced their speed earlier and experienced a greater decrease before entering signal-controlled intersections. This behavior can be attributed to their use of a self-regulation driving strategy. Self-regulation involves actively adjusting driving behavior, such as slowing down and increasing the distance, when drivers recognize a decline in their driving skills. This strategy ensures sufficient time to handle future traffic risks [29].

Starting from the turning point, older drivers began to increase their speed, while young and middle-aged drivers first reduced their speed and then increased it. This difference arose from older drivers’ earlier reduction in speed, allowing them to prepare for a left turn through the intersection. Additionally, older participants in this study had extensive driving experience, which made them more sensitive to risks in complex environments. Their experience enabled them to adjust their driving activities in advance, ensuring adequate time and distance to avoid potential crashes [23].

**In terms of lateral driving behavior**, In terms of lateral driving behavior, older drivers exhibited smaller peak steering wheel angles and a smoother overall turning process. With accumulated driving experience, a moderating effect exists between drivers’ psychological abilities and their driving performance [30]. Older drivers tended to be more cautious and conservative compared to younger drivers in the same traffic environment [31,32]. At unsignal-controlled intersections, drivers in both groups initiated their turns earlier than at signal-controlled intersections, with this effect being more pronounced for older drivers. This can be attributed to the greater traffic risk at unsignal-controlled intersections, which presents more challenges for older drivers [33].

As age increases, the time required for perception, judgment, and action significantly extends [34]. To improve driving safety, it is crucial to consider the sensory limitations of older drivers in vehicle design and development. Intelligent driving assistance systems, such as intersection risk warning and collision avoidance systems based on safety distance, can help older drivers perceive risks and respond promptly, enhancing driving stability and safety. In addition to active safety technologies, traffic safety management departments can implement early warning systems for traffic signs and markings at pre-turn starting points at unsignal-controlled intersections, which would better align with the cognitive habits of older drivers.

### Differences of different ages drivers in the spatial dimension

**In terms of longitudinal driving behavior**, older drivers reduced their speed earlier before entering signal-controlled intersections, and their speed fluctuated more frequently. This suggests that, despite taking preventive measures, older drivers still faced difficulties in perceiving and balancing vehicle control with risk levels [35]. The decline in perception and execution functions may lead to inaccurate driving judgments, requiring their driving behavior to be repeatedly corrected based on the results of vehicle operation. Additionally, older drivers’ tendency to minimize risks resulted in greater and more frequent fluctuations in their driving speed [36]. At unsignal-controlled intersections, both groups of drivers decelerated earlier, but the speed fluctuations of older drivers were relatively smaller. This suggests that early preparation allowed older drivers sufficient time to adjust, leading to smoother driving.

**In terms of lateral driving behavior,** older drivers initiated steering earlier and exhibited smaller fluctuations in steering angle. At unsignal-controlled intersections, both groups of drivers turned earlier, but older drivers not only initiated their turns earlier but also had larger steering wheel angles with more frequent fluctuations. This can be attributed to the greater risks older drivers face at intersections compared to younger drivers [37,38]. Although extensive driving experience can compensate for handling and control abilities [39], its impact is limited. The decline in sensory function still requires older drivers to continuously adjust their driving behavior to ensure safety.

Intersections require high visual attention and expose drivers to greater risks [38]. Older drivers tend to slow down and initiate steering earlier than younger drivers. However, they may still experience urgency and become hazardous due to frequent adjustments in their driving behavior. Therefore, it is essential to address the driving needs of older drivers by developing age-friendly vehicle technologies, such as active warning systems for complex intersections. Additionally, traffic safety management departments should offer targeted training and intervention to help older drivers improve their ability to identify and respond to driving risks based on their specific driving characteristics.

### Differences in the time and spatial dimensions

**In terms of longitudinal driving behavior**, The driving speed of older drivers before turning at unsignal-controlled intersections was higher than at signal-controlled intersections. After passing the turning point at the unsignal-controlled intersection, their speed increased, remaining higher than at the signal-controlled intersection. This may be because the traffic light served as a warning [40], and the lack of signals made driving feel less stressful. Additionally, their speed fluctuations at signal-controlled intersections were concentrated in the latter part of the turning preparation area, while at unsignal-controlled intersections, fluctuations occurred throughout the entire area. This suggests that the absence of signal-controlled traffic facilities negatively impacted the driving behavior of older drivers [41].

**In terms of lateral driving behavior,** older drivers began turning earlier at the pre-turn point of the unsignal-controlled intersection compared to the signal-controlled intersection. This aligns with their safer and more cautious driving style. After passing the unsignal-controlled intersection, older drivers reduced the steering angle earlier. Their steering wheel movements began to fluctuate in the later part of the turning preparation area at the unsignal-controlled intersection, with more frequent fluctuations during the process. This suggests that the lateral control stability of older drivers at unsignal-controlled intersections was poor. They needed to continuously adjust the steering to correct lateral displacement deviations [42].

The environment at the unsignal-controlled intersection negatively affected older drivers’ operations, as evidenced by frequent fluctuations in speed and steering wheel angle. Erratic driving in this context is likely to lead to risky behavior. Therefore, in complex environments such as low-grade intersections and construction areas, it is important to consider the placement of traffic lights, signs, and markings in the pre-turn section. Protective measures should be implemented to provide early warnings to drivers and improve driving stability. Additionally, steering assistance systems could be integrated into vehicles designed for older drivers to enhance lateral control stability.

### Analysis of factors influencing cognitive driving behavior

**In terms of speed standard deviation**, drivers exhibited a larger value at signal-controlled intersections, which aligns with Long’s findings [43]. This may be because signal-controlled intersections act as psychological cues for drivers, making them more aware of the need to slow down. Additionally, performance in visual-spatial abilities, as measured by TMT-A, was correlated with speed standard deviation. Drivers with poorer visual-spatial abilities may face greater difficulties in driving, leading to increased speed fluctuations [44]. Older drivers showed lower speed standard deviation at signal-controlled intersections compared to middle-aged and young drivers. This could be attributed to their self-regulation strategies, resulting in lower and more stable driving speeds. Moreover, drivers with mild cognitive impairment (TMT-A) exhibited lower speed standard deviation compared to those with more severe cognitive impairment. This suggests that better visual-spatial abilities, combined with the presence of signal-controlled facilities, contribute to more stable speed variations. At signal-controlled intersections, drivers with TMT-B cognitive impairment showed lower speed standard deviation, which may be due to their reduced cognitive flexibility, prompting them to adopt more cautious driving behaviors and anticipatory deceleration for safety [45].

**Regarding steering wheel cornering standard deviation**, drivers exhibited greater variability at signal-controlled intersections, likely due to the need for cautious left turns. Older drivers demonstrated higher steering wheel cornering standard deviation than middle-aged and young drivers, consistent with spatial observations. This could be due to the fact that turning requires quicker judgments and actions, which present greater challenges for older drivers [46]. At unsignal-controlled intersections, older drivers also showed higher steering wheel cornering standard deviation, further supporting this observation. At signal-controlled intersections, drivers with normal cognitive function (MMSE, TMT-A) and mild cognitive impairment (TMT-A) had lower steering wheel cornering standard deviation compared to those with more severe cognitive impairment. This suggests that better cognitive abilities and visual-spatial skills lead to improved steering control [47]. In comparison to middle-aged and young drivers, older drivers with normal cognitive function (MMSE) displayed lower steering wheel cornering standard deviation, likely reflecting their cautious driving style [48].

### Improvement suggestions

Under different traffic control conditions and cognitive ability levels, the driving performance of older drivers differs significantly from that of young and middle-aged drivers. Older drivers exhibited poorer performance at unsignal-controlled intersections. Those with cognitive impairments struggle more with lateral turning control. Optimizing and redesigning intersection facilities is essential to better accommodate older drivers. At unsignal-controlled intersections, traffic signs and road markings should be adjusted to meet their needs.

In traffic infrastructure, improving warning and guidance systems can help compensate for older drivers’ reduced perception. At unsignal-controlled intersections, adding dynamic light-emitting diode warning signs, vibrating pavement markings, or ground guidance arrows can help them recognize intersections earlier [11]. Enhancing traffic signs and markings can also be effective. Using larger, clearer fonts, a simpler layout, and high-reflective materials improves visibility in complex lighting conditions and shortens reaction times [49]. Converting high-risk unsignal-controlled intersections into roundabouts can further reduce cognitive load for older drivers when turning left or navigating intersections [50].

In driving assistance technology, intelligent systems can support aging drivers. Intersection risk warning systems using vehicle-to-everything technology can provide alerts for oncoming traffic and potential collisions. These systems can improve warning flexibility and simplify vehicle interfaces to counteract age-related driving declines [51]. Intelligent speed control systems can also help. They can provide dynamic speed limit recommendations at unsignal-controlled intersections or automatically adjust throttle response if a driver fails to slow down in time. This ensures smoother passage through intersections [52]. Steering assistance should also be optimized. Stability support can help older drivers when making multiple corrections. Adaptive steering systems can adjust feedback force based on driving habits. This makes low-speed turns easier and improves intersection maneuverability [53].

### Limitations

First, this study used a driving simulation experiment method. While it realistically simulates road and traffic environments, it may differ slightly from real-world conditions. Future research could include on-road experiments to validate these findings further. Second, the study primarily focused on changes in driving stability (speed and steering angles). Future research could consider evaluating additional dimensions to provide a more comprehensive perspective. Finally, this study did not explore the impact of individual differences (such as gender, health status, etc.) on older drivers’ behavior. Future research could consider these factors to better understand the driving characteristics of older drivers from diverse backgrounds.

## Conclusion

This study explored the driving behavior characteristics of both young and middle-aged drivers and older drivers. Two simulated risk scenarios were designed for the experiment: signal-controlled and unsignal-controlled intersections. Longitudinal and lateral driving behavior indicators were used to analyze differences across time and space. The main findings are as follows:

1. Time dimension: older drivers reduced speed earlier and showed greater variation before reaching signal-controlled intersections. They also accelerated and turned earlier after the turning point.
2. Spatial dimension: older drivers slowed down and showed speed instability before entering intersections, they used smaller steering angles at signal-controlled intersections. They showed more variation in speed and steering, with higher steering angles at unsignal-controlled intersections.
3. Intersection types: at unsignal-controlled intersections, older drivers drove faster and turned earlier. At unsignal-controlled ones, their fluctuations in speed and steering were continuous throughout the preparation phase.
4. Driving stability: Traffic control, driver type, and cognitive function significantly influenced driving stability. At signal-controlled intersections, these factors interacted strongly and had greater effects.

Due to physical limitations, older drivers often experience declines in perception and control. Future vehicle and infrastructure design should consider these traits. Intelligent, age-friendly solutions are needed to address safety risks caused by functional decline. This study offers guidance for developing supportive systems, facilities, and training tailored to older drivers.

## Supporting information

S1 File Sl Table: Mini mental state examination. S2 Fig: Trail making test.
(DOCX)
