# Supplementary material for: A study on driving behavior characteristics and influencing factors of older drivers at signal-controlled and unsignal-controlled intersections
Source: PLoS One. 2025 Jun 25;20(6):e0326696. doi: 10.1371/journal.pone.0326696 (PMC12192173; doi:10.1371/journal.pone.0326696)
Supplement: S1 File — Sl Table: Mini mental state examination. S2 Fig: Trail making test. (DOCX) [file pone.0326696.s001.docx]

**S1 Table. Mini mental State Examination.**

| Score | Maximum Score | Questions |
| --- | --- | --- |
|  | 5 | “What is the year? Season? Date? Day of the week? Month?” |
|  | 5 | “Where are we now: State? County? Town/city? School? Floor? ” |
|  | 3 | The examiner names three unrelated objects clearly and slowly, then asks the patient to name all three of them. The patient's response is used for scoring. The examiner repeats them until patient learns all of them, if possible. Number of trials: |
|  | 5 | “I would like you to count backward from 100 by sevens.” (93, 86, 79.72,65,...) Stop after five answers.  Alternative: “Spell WORLD backwards.” (D-L-R-O-W) |
|  | 3 | “Earlier I told you the names of three things. Can you tell me what those were?” |
|  | 2 | Show the patient two simple objects, such as a wristwatch and a pencil.  and ask the patient to name them. |
|  | 1 | “Repeat the phrase: 'No ifs, ands, or buts.” |
|  | 3 | “Take the paper in your right hand, fold it in half, and put it on the floor.(The examiner gives the patient a piece of blank paper.) |
|  | 1 | “Please read this and do what it says.” (Written instruction is “Close your eyes. ”) |
|  | 1 | “Make up and write a sentence about anything.”  (This sentence must contain a noun and a verb.) |
|  | 1 | “Please copy this picture. ” (The examiner gives the patient a blank piece of paper and asks him/her to draw the symbol below. All 10angles must be present and two must intersect.)  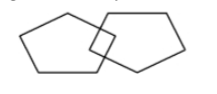 |


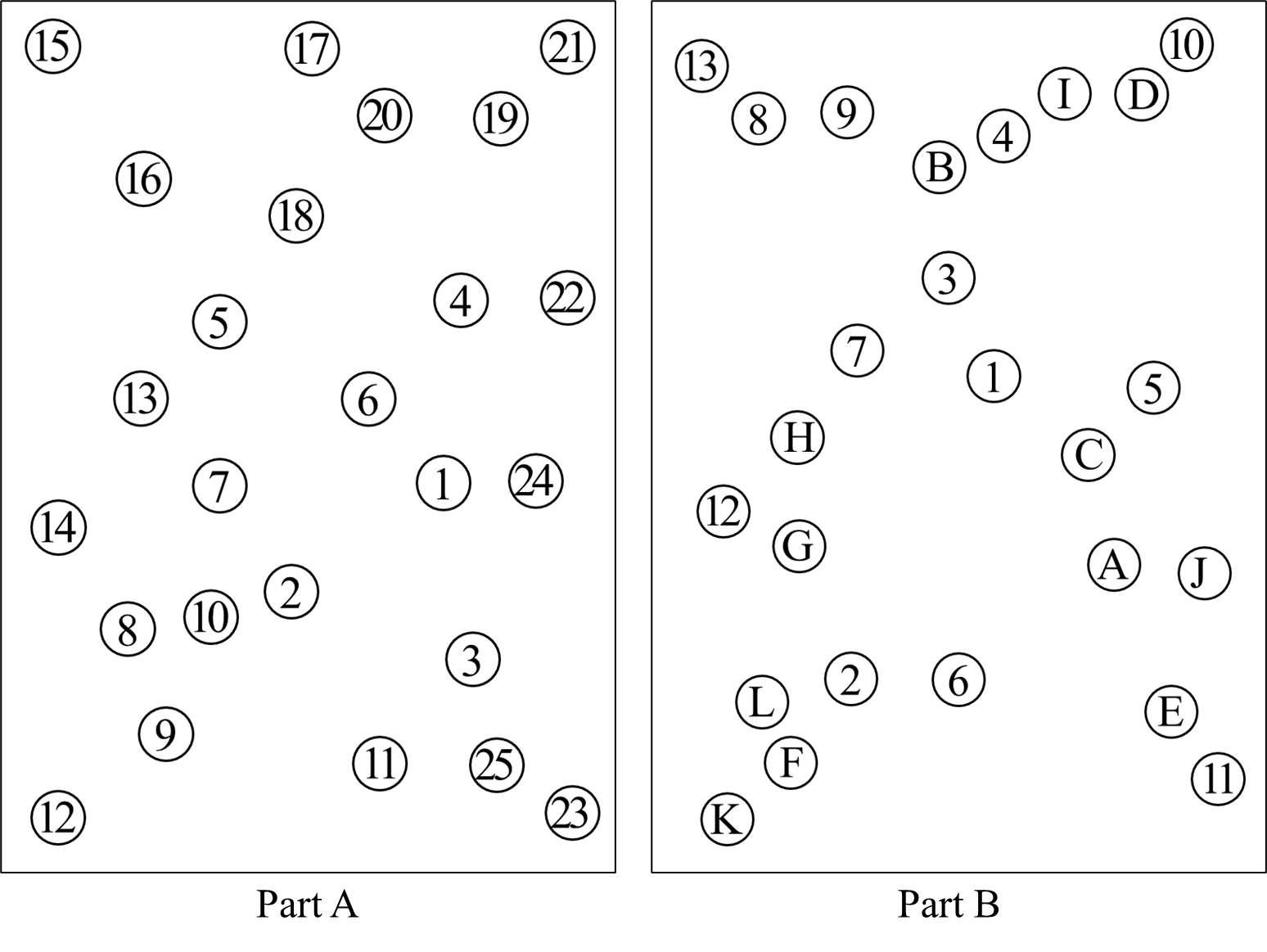


**S2 Fig. Trail Making Test.**
